# Comprehensive Multidisciplinary Management of Hyper-IgE Syndrome in an 11-Year-Old Female: A Pediatric Case Report

**DOI:** 10.7759/cureus.65377

**Published:** 2024-07-25

**Authors:** Ahmed Elashmawy, Mohammadali Chokr, Saima Sharif, Lauren Ferrantino

**Affiliations:** 1 Pediatrics, Wayne State University School of Medicine, Detroit, USA; 2 Anesthesia, Corewell Health East, Dearborn, USA; 3 Neonatology, Central Michigan University College of Medicine, Detroit, USA; 4 Pediatrics, Children's Hospital of Michigan, Detroit, USA

**Keywords:** job syndrome, hyper-ige syndrome, pediatric rare diseases, pediatric fractures, multi-disciplinary teams

## Abstract

Hyper-IgE syndrome (HIES) or Job syndrome is a rare immunodeficiency characterized by elevated levels of IgE and recurrent infections, eczema, and connective tissue abnormalities. Patients with HIES are prone to recurrent pyogenic and opportunistic infections due to impaired immune responses. Here, we present the case of an 11-year-old female diagnosed with HIES, who was admitted to the hospital with bacterial pneumonia and leg pain associated with a history of osteopenia. The patient’s clinical course included fever, cough, throat pain, and leg pain. Management involved a rigorous course of antibiotics, antifungals, and cultures of pertinent pathogens, along with imaging of the lower extremity. This case underscores the importance of appropriate management strategies for patients with HIES and their comorbidities to mitigate the risk of infections and improve patient outcomes.

## Introduction

Hyper-IgE syndrome (HIES) is a rare primary immunodeficiency disorder characterized by elevated levels of IgE in the presence of recurrent infections, eczema, and a range of other systemic manifestations. This condition, also known as Job syndrome, is associated with a defect in the immune system's ability to respond to infections and regulate allergic responses due to abnormally elevated IgE levels, which normally regulate allergic reactions through mast cell and basophil degranulation [1]. Job syndrome is characterized by a genetic mutation in the STAT3 gene, which impairs the function of T helper 17 (Th-17) cells, which are crucial for managing the body's response to certain pathogens and controlling inflammation [2].

The hallmark of HIES is the combination of recurrent staphylococcal infections, eczema, and elevated serum IgE levels. Patients are particularly susceptible to eczema similar to atopic dermatitis, bacterial and viral skin infections, cold abscesses, respiratory infections with possible pulmonary complications, allergies, gastrointestinal manifestations, malignancies, connective tissue abnormalities, chronic skin infections, sinusitis, and pneumonia due to defects in the immune system's ability to mount effective responses against pathogens [3,4]. The syndrome also often leads to skeletal and dental abnormalities, such as hyperextensible joints and retained primary teeth [5].

HIES presents with a variable clinical spectrum, including features of frequent, severe infections, and chronic eczema. The elevated IgE levels contribute to the heightened allergic responses and contribute to the chronic inflammatory state observed in these patients, with an increased incidence of anaphylaxis [6]. Diagnosis typically involves clinical assessment and confirmation through genetic testing to identify mutations associated with the syndrome.

Here, we present the case of an 11-year-old female diagnosed with HIES, who presented with cough, fever, sore throat, and leg pain associated with osteopenia. This case underscores the complexities of managing immunodeficiencies with elevated IgE and highlights the importance of early therapeutic interventions to mitigate infectious complications and optimize patient outcomes.

## Case presentation

Disclaimer: Ethics committee approval and patient consent were obtained and documented through the Wayne State University Institutional Review Board (approval no: 2024 142).

An 11-year-old female with a history of HIES (Job syndrome), osteopenia, and an extensive orthopedic history presented with a dry cough for three days and a fever of 100.2°F for one day. Her mother noted that the cough resembled previous episodes that progressed to pneumonia, but the patient was breathing without dyspnea or tachypnea. She also reported right leg pain. Her diagnosis of Job syndrome was made based on past immunological testing revealing a pathogenic mutation in the STAT3 gene. 

**Figure 1:**
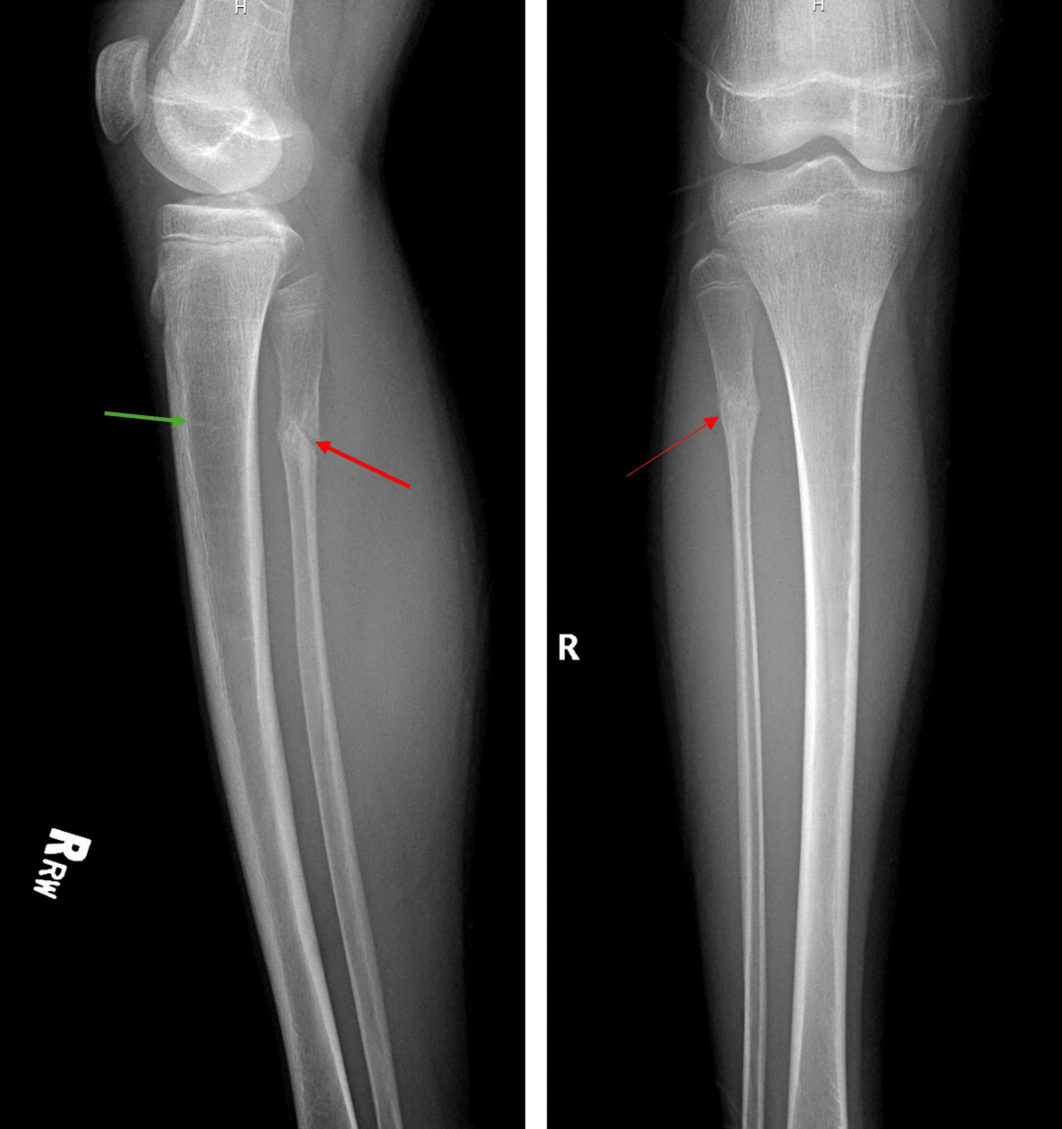
Frontal and lateral radiographs of the tibia and fibula X-ray reveals a subacute fracture at the proximal fibula diaphysis with early healing signs, chronic osteopenia, and multiple growth arrest lines. Osseous structures are normally aligned, and joints are stable with no acute soft tissue abnormalities.

The patient also has a past medical history of pulmonary aspergillosis diagnosed on bronchoalveolar lavage (BAL), recurrent pneumonia with a recent Pseudomonas infection, and a right lower lobe lobectomy. Additionally, she also has a history of multiple extremity fractures. Physical examination revealed right leg pain with deep tenderness to palpation, a full range of motion, and weight-bearing without a limp. Her knee was stable with no ligamentous laxity. X-rays showed a healed fracture of the fibula with no new acute fractures or obvious deformities (Figure 1). A possible occult fracture not visible on X-ray was considered, and an MRI was recommended if the pain persisted.

The X-ray also revealed persistent osteopenia, indicating reduced bone density. This condition is likely due to constant infections and the associated chronic inflammation that negatively impacts bone metabolism. Inflammatory cytokines can promote bone resorption and inhibit bone formation, leading to decreased bone density. The X-ray showed a transverse lucent line at the proximal fibula diaphysis, with mild sclerotic changes and a subtle periosteal reaction, suggesting a subacute fracture with early signs of healing. The bones were normally aligned with no abnormal angulation or displacement, and there was no soft tissue swelling, indicating no significant acute inflammatory response or injury. The knee joint was well-aligned with no dislocations or subluxations. Multiple growth arrest lines in the femur and proximal and distal tibia and fibula indicated periods of interrupted growth and recovery. Overall, the osseous structures were normally aligned, and the joints were stable with no acute soft tissue abnormalities.

**Figure 2:**
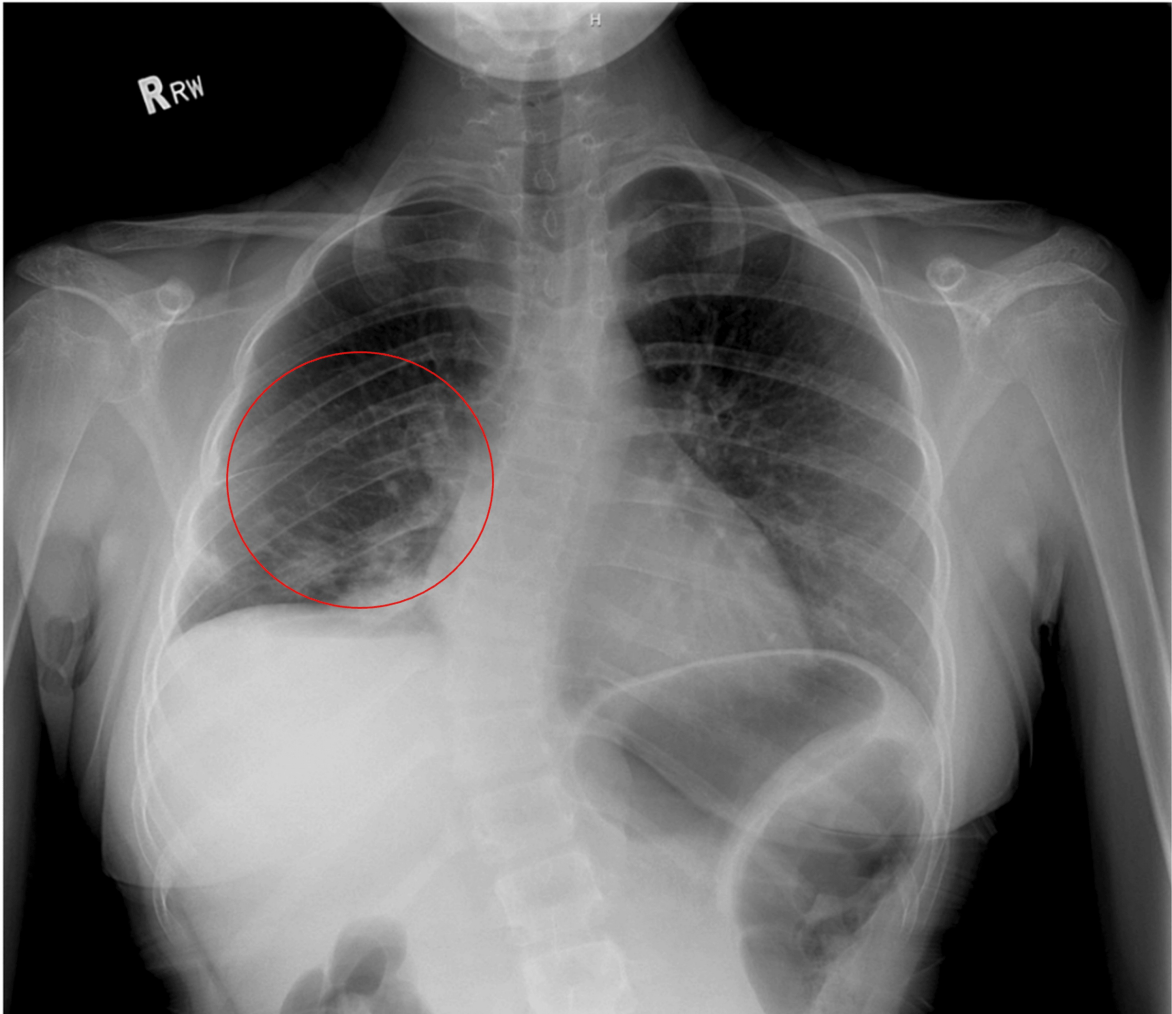
Radiograph of the chest X-ray indicates pneumonia in the right middle lobe, hypo-inflated lungs with post-surgical changes, no acute abnormalities like pleural effusion or pneumothorax, and a normal heart and mediastinum. Patchy airspace opacities in the right lower lung are outlined in a red circle.

The patient was admitted for the management of pneumonia. A chest X-ray is provided below (Figure 2). The X-ray reveals patchy airspace opacities in the right lower lung, indicating lung consolidation typical of pneumonia. The lungs are hypo-inflated, especially on the right side, which aligns with the patient's history of right lower lobectomy. There are new areas of patchy opacities in the right middle lobe. No pleural effusion or pneumothorax is observed. The cardiovascular silhouette appears normal in shape and size. Additionally, there is a curvature in the thoracolumbar spine, suggesting possible scoliosis or another spinal abnormality. In summary, the X-ray shows pneumonia in the right middle lobe, hypo-inflated lungs with post-surgical changes, no acute pleural effusion or pneumothorax, and a normal heart and mediastinum.

During the hospital course, she was monitored for fevers and respiratory status. She received four days of IV cefepime 2000 mg every 12 hours and four days of IV clindamycin 600 mg every eight hours for empiric coverage of gram-negative and gram-positive organisms, respectively. Itraconazole 200 mg daily was continued for fungal prophylaxis for four days as well, along with Bactrim prophylaxis as recommended by neurology. A serum galactomannan screen was sent to monitor for aspergillus infection, and a blood culture showed no growth for one day (Table 1). The comprehensive test results from the patient's recent assessment show a generally healthy profile with several notable findings. The patient tested negative for various respiratory viruses including Flu A, Flu B, RSV, and SARS-CoV-2, indicating an absence of some of the more common virus infections (Table 1 shows a list of pathogens tested).

**Table 1:** Comprehensive laboratory test results and diagnostic findings

Test Name	Result	Reference Range
Rapid Flu A by PCR POC2	Negative	Negative
Rapid Flu B by PCR POC2	Negative	Negative
Rapid RSV by PCR POC2	Negative	Negative
Rapid SARS (COVID-19) PCR POCT	Negative	Negative
Sodium	136 mMol/L	135-145 mMol/L
Potassium	3.4 mMol/L	3.5-5.0 mMol/L
Chloride	105 mMol/L	96-106 mMol/L
Carbon Dioxide	23 mMol/L	22-29 mMol/L
Anion Gap	8 mMol/L	3-11 mMol/L
Glucose	94 mg/dL	70-99 mg/dL
Urea Nitrogen (BUN)	9 mg/dL	6-20 mg/dL
Creatinine	0.55 mg/dL	0.6-1.2 mg/dL
Bilirubin - Total	0.28 mg/dL	0.1-1.2 mg/dL
Calcium	9.7 mg/dL	8.5-10.2 mg/dL
ALT (GPT)	12 Units/L	7-56 Units/L
AST (GOT)	16 Units/L	10-40 Units/Li
Alkaline Phosphatase	184 Units/L	44-147 Units/L
Total Protein	8.4 gm/dL	6.0-8.3 gm/dL
Albumin	4.6 gm/dL	3.5-5.0 gm/dL
eGFR CKD-EPI	Not calculated	>60 mL/min/1.73m²
WBC	7.9 K/CUMM	4.0-11.0 K/CUMM
RBC	4.05 M/CUMM	4.2-5.9 M/CUMM (men), 3.5-5.5 M/CUMM (women)
Hemoglobin	11.8 gm/dL	13.8-17.2 gm/dL (men), 12.1-15.1 gm/dL (women)
Hematocrit	35.6%	40.7-50.3% (men), 36.1-44.3% (women)
MCV	88.1 FL	80-100 FL
MCH	29.2 pg	27-33 pg
MCHC	33.2%	32-36%
Red Cell Distribution Width (RDW)	14.0%	11.5-14.5%
Platelets	269 K/CUMM	150-450 K/CUMM
Mean Platelet Volume (MPV)	7.5 FL	7.5-11.5 FL
Manual Diff Review	1	N/A
Absolute Lymphocyte Count	1.8 K/CUMM	1.0-3.0 K/CUMM
Lymphocyte %	23%	20-40%
Absolute Monocyte Count	0.7 K/CUMM	0.2-1.0 K/CUMM
Monocyte %	9%	2-8%
Absolute Neutrophil Count	4.8 K/CUMM	1.5-8.0 K/CUMM
Neutrophil %	62%	40-60%
Absolute Eosinophil Count	0.5 K/CUMM	0-0.5 K/CUMM
Eosinophil %	6%	1-4%
Absolute Basophil Count	0.0 K/CUMM	0-0.3 K/CUMM
Basophil %	0%	0-1%
Nucleated RBCs	0.0 K/CUMM	0 K/CUMM
Adenovirus	Not detected	Negative
Bordetella Pertussis	Not detected	Negative
Chlamydophila Pneumoniae	Not detected	Negative
Coronavirus (COVID-19) PCR - in house	Not detected	Negative
Coronavirus 229E	Not detected	Negative
Coronavirus HKU1	Not detected	Negative
Coronavirus NL63	Not detected	Negative
Coronavirus OC43	Not detected	Negative
Human Metapneumovirus	Not detected	Negative
Influenza A	Not detected	Negative
Influenza A 2009 H1	Not detected	Negative
Influenza A H1	Not detected	Negative
Influenza AH3	Not detected	Negative
Influenza B	Not detected	Negative
Mycoplasma Pneumoniae	Not detected	Negative
Parainfluenza 1	Not detected	Negative
Parainfluenza 2	Not detected	Negative
Parainfluenza 3	Not detected	Negative
Parainfluenza 4	Not detected	Negative
Para-pertussis PCR	Not detected	Negative
Resp PCR Spec Description	Nasal	N/A
Resp Syncytial Virus	Not detected	Negative
Rhinovirus/Enterovirus	Not detected	Negative

Key blood chemistry markers such as sodium, glucose, and calcium were within normal ranges. However, there were findings of low potassium levels and slightly elevated alkaline phosphatase and total protein levels. The patient received IV cefepime and IV clindamycin. Certain antibiotics can cause renal potassium loss or gastrointestinal loss, leading to hypokalemia. The patient was counseled on oral potassium supplementation in the future to offset this electrolyte abnormality. Hematological parameters revealed a low red blood cell count but a normal white blood cell count with balanced differential counts. These results collectively suggest a stable health condition with mild deviations in electrolytes and hematological indices that may warrant monitoring and follow-up.

## Discussion

In this case study, we presented an 11-year-old female with HIES, osteopenia, and a significant orthopedic history, who was admitted with symptoms of pneumonia and leg pain. The patient’s management included empiric antibiotic therapy, antifungal prophylaxis, and close monitoring, highlighting the complexities of treating patients with HIES.

Our key findings indicate that the patient's recurrent infections and osteopenia are significant challenges in managing HIES. These results align with the existing literature, which identifies recurrent staphylococcal infections and skeletal abnormalities as hallmark features of HIES [3-5]. The imaging studies confirmed pneumonia and a subacute fibular fracture, consistent with the expected complications in HIES patients [4].

Compared to previous studies, our findings underscore the necessity of a multidisciplinary approach to managing HIES. The combination of antibiotic and antifungal therapies, along with orthopedic and pulmonary consultations, was crucial in stabilizing the patient. This comprehensive care strategy is supported by similar approaches in other case studies, emphasizing the importance of addressing both infectious and non-infectious complications in HIES [4,7].

However, there are discrepancies in the frequency and severity of infections reported in different studies. Some studies report more frequent respiratory infections and severe immunodeficiency, while our patient exhibited a manageable condition with appropriate prophylaxis and treatment. These differences could be attributed to genetic variations, environmental factors, or differences in healthcare access and quality [8,9].

For this particular case, the use of a comprehensive antibiotics course was meant to ensure broad-spectrum coverage that would protect the patient from the multitude of bacterial pathogens that she is susceptible to due to her condition. Furthermore, the use of prophylactic antifungal therapy, as shown in our patient and her positive response to itraconazole, is essential to preventing opportunistic infections like aspergillosis. This approach is consistent with the literature recommendations, especially given the patient’s history of aspergillosis and the established guidelines on the use of antifungal therapy in immunocompromised patients [7,8]. Effective pain management is also critical to prevent complications such as respiratory atelectasis due to chest wall immobility from uncontrolled pain [9]. Atelectasis is a complication we wish to avoid in patients with respiratory symptoms like this, especially in those who are immunocompromised and prone to such infections. In regard to the patient's osteopenia and her frequent orthopedic problems, the administration of bisphosphonates may be considered in the future to minimize bone loss and reformation [10].

The implications of our findings for clinical practice are significant. They suggest that early diagnosis and a comprehensive, multidisciplinary treatment approach can effectively manage HIES, reduce the frequency of severe infections, and improve overall patient outcomes. Regular monitoring and proactive management of bone health are also critical to prevent and address osteopenia-related complications [5]. Additionally, the heightened allergic responses and chronic inflammatory state observed in these patients underscore the importance of comprehensive care [6].

For policy and further research, our findings highlight the need for guidelines on the multidisciplinary management of HIES. Future studies should focus on the long-term outcomes of different treatment regimens and the impact of early intervention on the quality of life in HIES patients. Additionally, research into the genetic and molecular basis of HIES could provide insights into targeted therapies and personalized treatment plans [2,5]. Our study adds to the understanding of HIES management by demonstrating the effectiveness of a multidisciplinary approach. These findings could influence current practices by promoting comprehensive care strategies and informing future research directions in the field of immunodeficiency disorders.

## Conclusions

This case highlights the intricacies of caring for pediatric patients with HIES. In this particular case, the syndrome was complicated by osteopenia and a history of orthopedic issues. Our patient was an 11-year-old female who presented with symptoms of pneumonia and right leg pain. Chest and lower extremity X-rays demonstrated bacterial pneumonia and a subacute fracture of the proximal fibula, respectively. Our treatment plan included empiric antibiotic therapy, antifungal prophylaxis, and close monitoring. A multidisciplinary approach, including pulmonary and orthopedic management, proved beneficial in stabilizing this patient’s condition. We strongly believe that this approach can certainly help other patients with rare immunodeficiencies such as Job syndrome.
